# Differentiation of Adipose Tissue Mesenchymal Stem Cells into Endothelial Cells Depends on Fat Depot Conditions: Regulation by miRNA

**DOI:** 10.3390/cells13060513

**Published:** 2024-03-14

**Authors:** Gemma Arderiu, Anna Civit-Urgell, Alberto Díez-Caballero, Fabrizio Moscatiello, Carlos Ballesta, Lina Badimon

**Affiliations:** 1 Cardiovascular-Program, Institut de Recerca Sant Pau, IIB-Sant Pau, 08025 Barcelona, Spain; acivit@santpau.cat (A.C.-U.); lbadimon@santpau.cat (L.B.); 2 Ciber CV, Instituto Carlos III, 28029 Madrid, Spain; 3 Faculty of Pharmacy and Food Science, University of Barcelona (UB), 08028 Barcelona, Spain; 4 Centro Médico Teknon, Grupo Quiron Salut, 08022 Barcelona, Spain; adiez-caballero@quirurgica.com (A.D.-C.); fabriziomoscatiello@gmail.com (F.M.); info@clb.es (C.B.)

**Keywords:** adipose tissue, miRNAs, angiogenesis, ASCs, endothelial cells

## Abstract

The development of obesity is associated with substantial modulation of adipose tissue (AT) structure. The plasticity of the AT is reflected by its remarkable ability to expand or reduce in size throughout the adult lifespan, which is linked to the development of its vasculature. This increase in AT vasculature could be mediated by the differentiation of adipose tissue-derived stem cells (ASCs) into endothelial cells (ECs) and form new microvasculature. We have already shown that microRNA (miRNA)-145 regulates the differentiation of ASCs into EC-like (ECL) cells. Here, we investigated whether ASCs-differentiation into ECs is governed by a miRNAs signature that depends on fat depot location and /or the metabolic condition produced by obesity. Human ASCs, which were obtained from white AT by surgical procedures from lean and obese patients, were induced to differentiate into ECL cells. We have identified that miRNA-29b-3p in both subcutaneous (s)ASCs and visceral ASCs and miRNA-424-5p and miRNA-378a-3p in subcutaneous (s)ASCs are involved in differentiation into EC-like cells. These miRNAs modulate their pro-angiogenic effects on ASCs by targeting *FGFR1*, *NRP2*, *MAPK1*, and *TGF-β2*, and the MAPK signaling pathway. We show for the first time that miRNA-29b-3p upregulation contributes to ASCs’ differentiation into ECL cells by directly targeting *TGFB2* in both sASCs and visceral ASCs. Moreover, our results reveal that, independent of sASCs’ origin (obese/lean), the upregulation of miRNA-378a-3p and the downregulation of miRNA-424-5p inhibit *MAPK1* and overexpress *FGFR1* and *NRP2*, respectively. In summary, both the adipose depot location and obesity affect the differentiation of resident ASCs through the expression of specific miRNAs.

## 1. Introduction

The prevalence of obesity has risen rapidly worldwide. Obesity is associated with the leading causes of death worldwide, including diabetes, cardiovascular diseases, stroke, and several site-specific cancers [1]. Unlike most other tissues, adipose tissue (AT) continuously undergoes expansion and regression throughout adult life. AT consists of mature adipocytes surrounded by a stromal–vascular cell fraction (SVF) containing preadipocytes, endothelial cells (ECs), pericytes, fibroblasts, macrophages, and mesenchymal stem cells (adipose-derived stem cells [ASCs]) surrounded by a well-defined vascular system. AT expansion requires continuous remodeling of the vascular network, formation of new capillaries (for adipocyte hyperplasia), or remodeling from existing capillaries (for adipocyte hypertrophy) [2,3].

ASCs stimulate angiogenesis through the secretion of growth factors, cytokines, extracellular vesicles, and pro-angiogenic molecules that participate in the formation of neovascular-like structures interacting with microvascular ECs [4,5,6,7,8,9,10,11]. Moreover, ASCs may differentiate into EC-like (ECL) cells to form new blood vessels.

Importantly, fat depot localization seems to impact the resident ASCs’ proliferative capacity and their potential to differentiate into cells of the adipogenic lineage [12,13]. ASCs in subcutaneous AT have a higher ability to proliferate and differentiate than those obtained from visceral AT [13]. Moreover, different gene expression profiles have been identified in ASCs from subcutaneous AT and visceral AT. In subcutaneous AT, ASCs overexpress genes involved in proliferation, whereas in visceral AT, ASCs enhance gene clusters related to lipid biosynthesis and metabolism and downregulate genes associated with adipocyte differentiation and proliferation [13]. In addition, it has been demonstrated that obesity exerts a negative influence on AT functions [14]. AT from obese subjects has a significantly lower number of stem cells with reduced differentiation potential and is unable to form enough tube-like structures under angiogenic stimuli [15]. Moreover, ASCs derived from obese subjects present a reduced ability to migrate and invade, together with reduced angiogenic and growth potential [16]. Differences in cell surface marker expression, exosome content, percentage of senescent cells, and transcriptomic profile have also been demonstrated [14].

ASCs have the ability to differentiate into preadipocytes, chondrocytes, and osteoblasts, both in vitro and in vivo [11]. Likewise, there are numerous reports on ASCs’ differentiation into other cell types: hepatocytes [17], neuronal cells [18], cardiomyocytes [19], and ECs [20,21]. Induction of ASC differentiation into ECL cells in vitro requires the presence of high concentrations of growth factors, 3D cultures, or cocultures with other cell lines to induce lineage-specific differentiation [22,23].

MicroRNAs (miRNAs) are single-stranded short noncoding RNA molecules (approximately 20–25 nucleotides) that can bind to complementary target sites in mRNA molecules and repress translation or reduce mRNA stability, consequently blocking their translation into proteins [24]. The term angio-miR emerged to identify specific miRNA signatures involved in angiogenesis regulation and vessel remodeling [25]. Some of the angio-miRNAs identified are: miRNA-126, miRNA-145, miRNA-21, and miRNA-31. In obese patients, the expression of miRNA-126 in microvesicles secreted by ASCs is reduced compared to that in lean subjects. This miRNA reduction resulted in microvesicles that are biologically inactive for new vessel formation [26,27]. miRNA-31 expression was found to be upregulated in visceral AT from obese and type 2 diabetes mellitus patients compared to healthy subjects [28]. miRNA-21 seems to activate TGF-β signaling, which contributes to ASCs’ differentiation into adipocytes [29]. On the other hand, the inhibition of miRNA-145 has been shown to regulate ASCs’ differentiation toward ECs through the regulation of the ETS1 transcription factor [30].

Here, we hypothesize that the fat location and the metabolic state of a patient due to obesity may influence the differentiation capacity of ASCs into ECL cells to form micro-vessel networks through epigenetic-miRNA controlled mechanisms. Understanding these mechanisms may be used as future therapeutic targets to control the expansion and development of AT vasculature and obesity.

## 2. Materials and Methods

### 2.1. ASC Isolation and Characterization

To prepare the ASC cultures, AT was obtained via surgical resection from young individuals with morbid obesity (BMI > 40 kg/m^2^) who underwent bypass gastric surgery. AT was obtained simultaneously from subcutaneous and visceral fat depots from the same subject. Additionally, we collected AT from young individuals with normal weight (BMI ≤ 25 kg/m^2^) who underwent abdominal lipectomy. Samples were kept as a biological repository approved by the Hospital de Sant Pau Ethical Committee (Colección 01/2020). The tissue samples were anonymized when passed from the surgical room to the laboratory technician in charge of taking the tissue to the cell culture facilities. Another code was provided when entering the tissue culture facility. Therefore, no possible relation from the tissue to the donor could be established. The isolated cells in passage 1 were kept frozen until used. Information on a minimal set of clinical parameters is associated with the code. Information on the cells used in this study is presented in Supplemental Table S1.

Isolation of primary human ASCs was performed, as previously described in Arderiu et al. [26]. Briefly, subcutaneous and visceral AT were washed with sterile phosphate buffered saline supplemented with 100 U/mL penicillin and 100 μg/mL streptomycin (Gibco, Life Technologies, Grand Island, NY, USA). The tissue was digested by a 1 mg/mL type I collagenase solution (Sigma-Aldrich, St. Louis, MO, USA) and incubated for 1 h in a 37 °C prewarmed orbital shaker. Collagenase activity was neutralized with the same amount of fetal bovine serum (FBS) (Biological Industries, Kibbutz Beit-Haemek, Israel), and the suspension was filtered through a 100 μm mesh filter (Corning Costar Corp., Corning, NY, USA) to eliminate remaining tissue fragments. Then, the solution was centrifuged at 1200 rpm for 10 min to obtain the adipocyte-containing SVF collected from the pellet. Isolated SVF cells were counted and either analyzed by flow cytometry or plated onto 25 cm^2^ culture flasks (TPP, Reactiva, Trasadingen, Suwitzerland). After 24 h, nonadherent cells were removed, and the medium was replaced. Cells were expanded in a humidified environment at 37 °C with 5% CO_2_ and maintained at subconfluent levels prior to phenotypic analysis. The identity of ASCs was defined using the following criteria: adherence to plastic, cell surface antigen phenotyping, and differentiation into multiple cell lineages, as described in Oñate et al. [31]. CD105, CD90, CD29, CD44, CD45, and CD34 were determined. Cells were cultured in Dulbecco’s Modified Eagle’s Medium (DMEM)-Low Glucose (Gibco) supplemented with 10% FBS, 100 U/mL penicillin, and 0.1 mg/mL streptomycin. ASCs were used between passages 2 and 4.

### 2.2. MTS Viability/Proliferation Assay

Cell proliferation was determined by a 3-(4,5-dimethylthiazol-2-yl)-5-(3-carboxymetho-xyphe-nyl)-2-(4-sulfopheny)-2H-tetrazolium (MTS) Cell Proliferation Assay kit (colorimetric) (ab197010) (Abcam, Cambridge, UK). For this assay, 1 × 10^3^ cells were seeded in quadruplicate into a 96-well microtiter plate (Corning Costar Corp.). ASCs were cultured for 24 h with DMEM or DMEM supplemented with 10% FBS, 100 U/mL penicillin, 0.1 mg/mL streptomycin, and 10 ng/mL bFGF (233-FB, R&D Systems, Minneapolis, MN, USA). Finally, 20 μL of MTS per well were added and incubated for an additional 4 h, while MTS tetrazolium was reduced to formazan (490 nm absorbance) by metabolically active cells. The absorbance was then quantified with the spectrophotometer Spectramax 250 and analyzed with SoftMax software v2.0.16 (Molecular Devices, San Jose, CA, USA). Formazan production was directly related to the number of cells alive in the culture.

### 2.3. Induction of Endothelial Differentiation and Characterization

To induce ASC differentiation into ECL cells, ASCs were cultured in DMEM-Low Glucose supplemented with 10% FBS, 100 U/mL penicillin, 0.1 mg/mL streptomycin 10 ng/mL, and bFGF. Cells were supplied with fresh medium every three days and cultured for a total of nine days. ECL differentiation was confirmed by the expression of vascular EC markers, as described in Arderiu et al. [30]. Briefly, RNA from cell lysates was extracted by a RNeasy isolation kit (Qiagen, Valencia, CA, USA) and reverse transcribed by a High-Capacity cDNA Archive Kit (4368812, Applied Biosystems, Foster City, CA, USA). TaqMan qRT-PCR was run on the ABIPRISM 7900HT Fast Real-Time PCR System (Applied Biosystems), and the analysis was performed using SDS 2.4 software (Applied Biosystems). Transcript levels of EC markers were measured for PECAM-1 (Hs01065282m1), VE-cadherin (Hs00901465m1), vWF (Hs01182962m1), and eNOS (Hs01574659m1) (Applied Biosystems). The housekeeping gene GAPDH (Hs02786624_g1) (Life Technologies, Madrid, Spain) was used to normalize the results.

### 2.4. miRNA Isolation and Quantification

After endothelial induction for nine days, cells were harvested, and miRNA was isolated using a mirVana™ miRNA Isolation Kit with phenol (Life Technologies), according to the manufacturer’s instructions. cDNA was synthesized by the TaqMan™ Advanced miRNA cDNA Synthesis Kit (Life Technologies). An ND-1000 ultraviolet spectrophotometer (Nanodrop Technologies, Wilmington, DE, USA) was used to quantify the extracted miRNA.

### 2.5. miRNA 4.0 Affymetrix Library Preparation and Sequencing

Five hundred nanograms from each extracted miRNA sample were analyzed using GeneChip miRNA 4.0 arrays (Thermo Fisher Scientific). Samples were processed according to the manufacturer’s instructions with minimal modifications using the FlashTag Biotin HSR RNA Labeling Kit (Thermo Fisher Scientific) and the GeneChip Scanner 3000 7G System (including the GeneChip Scanner 3000 7G, Fluidics Station 450, and Hybridization Oven 645). Briefly, a poly-A tail was incorporated into RNA, followed by a ligation reaction. Every biotin-labeled RNA sample was hybridized into GeneChip miRNA 4.0 array cartridges and detected with avidin–streptavidin–phycoerythrin conjugate, which binds to biotin-labeled RNA. Hybridization was performed by incubating miRNA array cartridges at 48 °C with 60 rpm rotation for 18 h. Upon hybridization, each array was washed and stained using the FS450_002 fluidics script. Arrays were scanned, and data were exported for further analysis.

### 2.6. miRNA Data Analysis

A total of 4603 sequences were analyzed using miRNA arrays in human subcutaneous (s)ASCs from lean subjects and sASCs/ visceral (v)ASCs from obese subjects under undifferentiated and ECL cell differentiation conditions. Specifically, we analyzed the expression of 2025 pre-miRNAs and 2578 mature miRNAs under each condition. The results of the microarray data allowed us to differentiate between upregulated and downregulated miRNAs in ASCs from the different fat depots. MiRNAs with a *p*-value < 0.05 were considered significant for further analysis. MiRNAs with a log_2_ (fold change (FC)) ±6 were considered either upregulated or downregulated.

### 2.7. qRT-PCR Analysis of miRNA Expression

To validate the regulation of different miRNAs, TaqMan real-time quantitative reverse–transcriptase polymerase chain reaction (qRT-PCR) was run on the ABIPRISM 7900HT Fast Real-Time PCR System (Applied Biosystems), and the analysis was performed using SDS 2.4 software (Applied Biosystems). MiRNA levels were quantified using hsa-miRNA-424-5p (000604), hsa-miRNA-378a-3p (000567), hsa-miRNA-29b-3p (000413), hsa-miRNA-27b-5p (002174), hsa-miRNA-146a-5p (000468), hsa-miRNA30c-2-3p (002110) and hsa-miRNA-214-5p (002293), and internal normalized control hsa-miRNA-186-5p (002285) (Applied Biosystems). The results were determined and normalized to the reference miRNA hsa-miRNA-186-5p.

### 2.8. Integrated Target Prediction of Differentially Expressed miRNAs and Enrichment Analysis

Bioinformatic prediction of potential target genes and miRNA binding sites was performed using TarBase v8.0 available at “https://carolina.imis.athena-innovation.gr/ (accessed on 12 November 2021)”, TargetScan v7.2 available at “http://www.targetscan.org/ (accessed on 12 November 2021)”, MiRWalk v3.0 available at “http://mirwalk.umm.uni-heidelberg.de/ (accessed on 12 November 2021)”, and miRDB v6.0 available at “http://mirdb.org (accessed on 12 November 2021)”. Only the target genes predicted by all software programs were considered for further analysis. Differentially expressed Gene Ontology (GO) classes and Kyoto Encyclopedia of Genes and Genomes (KEGG) pathway enrichment analysis were applied using The Database for Annotation, Visualization and Integrated Discovery (DAVID) Bioinformatics Resources, available at “https://david.ncifcrf.gov/home.jsp (accessed on 18 January 2022)”. *p*-values < 0.05 were considered significant.

### 2.9. miRNA or Anti-miRNA Transfection

miRNAs and miRNA inhibitors were transfected into ASCs in 6-well plates at the following concentrations: miRNA-29b-3p mimic (50 nM) (MC10432), miRNA-378a-3p mimic (50 nM) (MC11360), miRNA-424-5p inhibitor (200 nM) (MH10306), or 50 nM mirVana negative control mimic/inhibitor (AMBION by Life Technologies Corporation) in each respective ASC sample. Cells were transfected using Lipofectamine RNAiMAX reagent (Life Technologies Corporation), according to the manufacturer’s protocol.

### 2.10. RNA Isolation and qRT-PCR of Target Genes

To assess the levels of regulated target genes, RNA from cell lysates was extracted by an RNeasy isolation kit (Qiagen) and reverse transcribed by a High-Capacity cDNA Archive Kit (4368812, Applied Biosystems). TaqMan qRT-PCR was run on the ABIPRISM 7900HT Fast Real-Time PCR System (Applied Biosystems), and the analysis was performed using SDS 2.4 software (Applied Biosystems). RNA levels were quantified using fibroblast growth factor receptor 1 (*FGFR1*) (Hs00241111_m1), mitogen-activated protein kinase 1 (*MAPK1*) (Hs01046830_m1), neuropilin 2 (*NRP2*) (Hs00187290_m1), and transforming growth factor-beta 2 (*TGF-β2*) (Hs00234244_m1). The results were determined and normalized to the reference gene *GAPDH* (Hs02786624_g1) (Thermo Fisher Scientific).

### 2.11. Constructs and Plasmids

The human *FGFR1* 3′-untranslated region (UTR) (136 bp) and *NRP2* 3′-UTR (270 bp), which included the predicted miRNA-424-5p seed sequences (TargetScan, miRDB), the *MAPK1* 3′-UTR (139 bp) that included the predicted miRNA-378a-3p seed sequence, and the *TGF-β2* 3′-UTR (177 bp) that included the predicted miRNA-29b-3p seed sequence, were amplified from genomic DNA by PCR and confirmed by sequencing. The following oligonucleotides were used (restriction digestion sites are underlined):

*FGFR1:* FOR-5′-CCCACAGAGCTCGTCGTTACCAGAGATTTACCCA-3′ and

REV-5′-GGAGGTCTAGAGGACATTCCCACCCTTTTCA;

*NRP2:* FOR-5′-CCC ACAGAGCTCTCAAAGGGAGGCATCAGGAA-3′ and

REV-5′-GGAGGTCTAGACATATTAACGCCTAAGGATTGCC-3′;

*MAPK1:* FOR-5′-CCCACAGAGCTCCGGTTTCTGGTAGTTGTGGC-3′ and

REV-5′-GGAGGTCTAGATGGTTTGAAAGATGCAGTGGT-3′;

*TGF-β2:* FOR-5′-CCCACAGAGCTCCAATTTGATCGTTGGCATGGT-3′ and

REV-5′-GGAGGTCTAGACTCGATGATGGTACTGATAGGA-3′; and cloned and inserted into the pmirGLO vector (Promega, Madison, WI, USA). CTGCT in two predicted seed sequences of *FGFR1* and three predicted seed sequences of *NRP2* were mutated to TAATACT, AGTCCAGA in the predicted seed sequence of *MAPK1* was mutated to AACGTCGA, and TGGTGCT in the predicted seed sequence of *TGF-β2* was mutated to GTAATAAT.

### 2.12. Luciferase Reporter Assay

An immortalized human epithelial cell line (HeLa) and human embryonic kidney (HEK) 293 cells from the Center for Disease Control (Atlanta, GA, USA) were routinely maintained in DMEM-High glucose media (Thermo Fisher Scientific) supplemented with 10% FBS, 100 U/mL penicillin, 0.1 mg/mL streptomycin, and 1% sodium pyruvate (Thermo Fisher Scientific).

To analyze the effects of miRNA-424-5p on 3′-UTR-*FGFR1*, 3′-UTR-*NRP2* miRNA-378a-3p on 3′-UTR-*MAPK1*, and miRNA-29b-3p on 3′-UTR-*TGF-β2*, HeLa or HEK293 cells were co-transfected with 50 ng of pmirGLO empty vector, pmirGLO wild-type *FGFR1*, *NRP2*, *MAPK1*, or *TGF-β2* 3′-UTR, or pmirGLO-mutated *FGFR1*, *NRP2*, *MAPK1,* or *TGF-β2* 3′-UTR vector, along with hsa-miRNA-424-5p mimic (MC10306), hsa-miRNA-424-5p inhibitor, hsa-miRNA-378a-3p mimic, hsa-miRNA-378a-3p inhibitor (MH11360), or hsa-miRNA-29b-3p mimic, hsa-miRNA-29b-3p inhibitor (MH10103), or a negative control at a final concentration of 50 nM, using the Lipofectamine 2000 assay kit (Invitrogen, Carlsbad, CA, USA). Thirty-six hours after transfection, cells were lysed with 1X passive lysis buffer, and Renilla luciferase activity was measured using the Dual Luciferase Assay kit (Promega) in cell extracts using a luminometer Infinite 200Pro (Tecan, Barcelona, Spain) following the manufacturer’s protocol. The results were normalized to Renilla luciferase activity (relative luciferase units, RLU) and referred to the pmirGLO empty vector as a control. Experiments were performed at least three times in triplicate to obtain statistical significance.

### 2.13. Statistical Analysis

Statistical calculations were performed on at least three independent experiments for each treatment. For in vitro experiments with more than six experiments, data normality was calculated with the Shapiro–Wilk test to prove Gaussian distribution. Values were tested for significant differences by using the parametric analysis of two-tailed Student’s *t* test, Wilcoxon matched-pairs signed rank test, or ANOVA for independent measures and the Tukey post hoc test when normally distributed. For not normally distributed data, the nonparametric analysis of variance U de Mann–Whitney or H–Kruskal–Wallis test for independent measures was used. The statistical software package GraphPad Prism version 8.0.0 (GraphPad Software Inc., San Diego, CA, USA) was used for statistical analyses. The results are expressed as the mean ± standard deviation (SD), and the number of experiments is indicated in the figure legends. Differences were considered statistically significant when *p* < 0.05.

## 3. Results

### 3.1. bFGF Induces Subcutaneous ASCs and Visceral ASCs to Differentiate into ECL Cells

**Figure 1:**
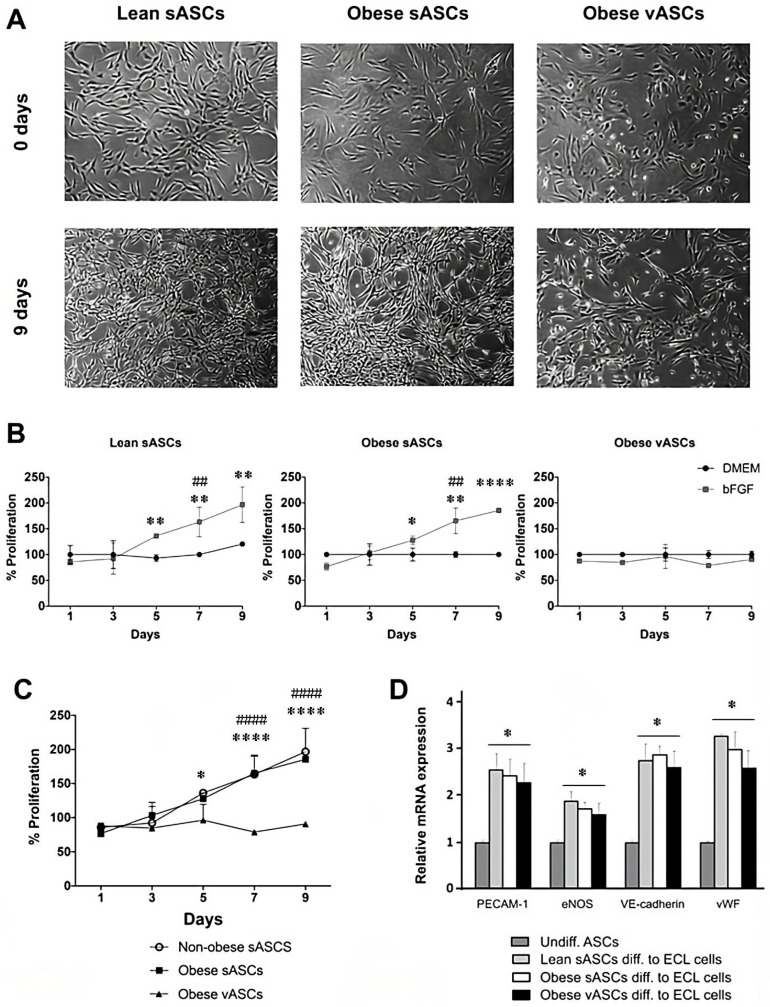
Characterization of the dynamic process of ASCs’ differentiation into ECL cells. ASCs were cultured with 10 ng/mL bFGF for nine days, and the medium was replaced every three days. (**A**) sASCs from lean and obese subjects and vASCs of the same obese subject in an undifferentiated state and after nine days of culture with DMEM supplemented with bFGF (Magnification 4×). (**B**) MTS cell proliferation assay of lean sASCs and obese sASCs and vASCs cultured with DMEM or DMEM supplemented with bFGF on days 1, 3, 5, 7, and 9. Results are shown as the arithmetic mean ± SD of four independent experiments, and statistical analysis was performed by the Wilcoxon matched-pairs signed rank test. * *p*-value < 0.05, ** *p*-value < 0.01, **** *p*-value < 0.0001 bFGF vs. DMEM, and ## *p*-value < 0.01 Day 1 bFGF vs. Day 7 bFGF (*n* = 4). (**C**) MTS cell proliferation assay of lean sASCs and obese sASCs and vASCs cultured with DMEM supplemented with bFGF on days 1, 3, 5, 7, and 9. Data represent the mean ± SD of four independent experiments, and statistical analysis was performed by two-way ANOVA followed by Tukey’s post hoc test. * *p*-value < 0.05, **** *p*-value < 0.0001 obese sASCs vs. vASCs, and #### *p*-value < 0.0001 lean sASCs vs. vASCs (*n* = 4). (**D**) mRNA expression of vascular EC markers after nine days of culture with 10 ng/mL bFGF. Results are shown as the arithmetic mean ± SD of six independent experiments, and statistical analysis was performed by two-way ANOVA followed by Tukey’s post hoc test. * *p*-value < 0.05 Undiff ASCs vs. Diff ASCs to ECL cells (*n* = 6).

To characterize the dynamics of ASCs’ differentiation into ECL cells, we determined ASCs’ morphology, proliferation, and expression of vascular EC markers. ASCs were cultured in DMEM supplemented with 10 ng/mL bFGF for nine days (Figure 1A). ASCs changed to a spindle-shaped morphology and reduced their size after five days with bFGF. The MTS assay showed significant differences in growth rates between DMEM and DMEM-bFGF ASCs cultures over the nine-day treatment. In sASCs from lean and obese subjects, bFGF significantly induced increased growth on day 5 (*p*-value < 0.01 and *p*-value < 0.05, respectively) but not in vASCs (a mild inhibitory effect was seen on day 7) (Figure 1B). Moreover, both lean and obese sASCs reached confluence faster in DMEM supplemented with bFGF than in DMEM culture medium. In addition, the MTS assay demonstrated that vASCs from obese individuals had slower growth than sASCs from both lean and obese subjects (Figure 1C). ASCs’ acquisition of the ECL phenotype was further assessed by evaluating PECAM-1, VE-cadherin, vWF, and eNOS transcript levels using qRT-PCR. ASCs differentiated into ECL cells significantly expressed vascular EC markers (*p*-value < 0.05), but undifferentiated ASCs did not (Figure 1D).

### 3.2. Differential miRNA Profile in ASCs from Different Fat Depots and Metabolic Conditions after Their Differentiation into ECL Cells

**Figure 2:**
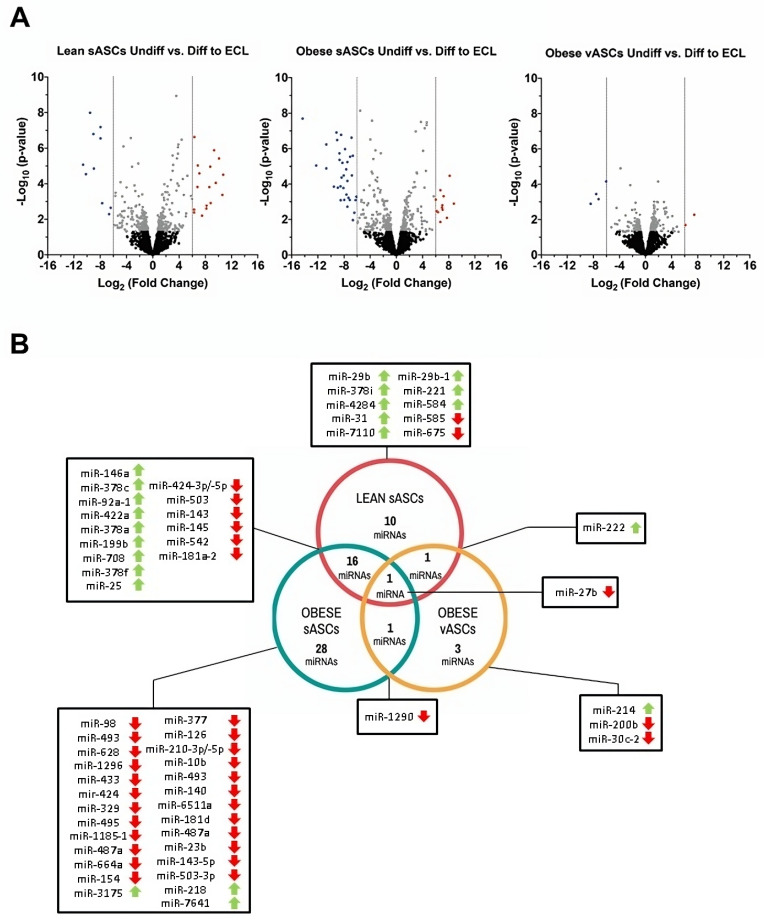
Differentiation into ECL cells modifies the miRNA profile of ASCs (nine days in culture with DMEM supplemented with bFGF). (**A**) Volcano plots of differentially expressed miRNAs in sASCs from lean donors and sASCs and vASCs from the same obese individual differentiated into ECL cells with a significance threshold of ±6-fold (*p*-value < 0.05). (**B**) Venn diagram showing the number of miRNAs differentially expressed in ASCs differentiated into ECL cells.

Arrays were run on ECL cells differentiated from the three types of ASCs after nine days in bFGF-DMEM culture. The expression patterns of the miRNAs obtained are shown in volcano plots (Figure 2A). In ECL cells differentiated from sASCs of lean subjects, we found 18 miRNAs significantly upregulated and 10 significantly downregulated with respect to the undifferentiated sASCs (Figure 2A). Among these, miRNA-424 and miRNA-29b were present in both arms (-3p and -5p). The top five most significantly regulated miRNAs were miRNA-29b-3p (10.7-fold), miRNA-146a-5p (10.6-fold), miRNA-181a-2-3p (-10.6-fold), miRNA-542-5p (-10.2-fold), and miRNA-378c (10.1-fold) (Supplemental Table S2). In contrast, in ECL cells differentiated from sASCs of obese individuals, 12 miRNAs were significantly upregulated, and 34 were significantly downregulated (Figure 2A). In the following miRNAs, both arms were involved in endothelial differentiation: miRNA-424, miRNA-143, miRNA-493, miRNA-503, and miRNA-210. Notably, miRNA-424 was also represented by its immature sequence (Supplemental Table S3). The top five downregulated miRNAs were miRNA-181a-2-3p (-14.3-fold), miRNA-1290 (-12.2-fold), miRNA-542-5p (-10.7-fold), miRNA-424-5p (-10.7-fold), and miRNA-23b-5p (-9.5-fold) (Supplemental Table S3). Finally, in ECL cells differentiated from the vASCs of obese individuals (from the same obese donors of sASCs), only two miRNAs were significantly overexpressed, and four miRNAs were significantly downregulated (Figure 2B): miRNA-214-5p (up, 7.5-fold), miRNA-222-5p (up, 6.1-fold), miRNA-1290 (-8.4-fold), miRNA-30c-2-3p (-7.6-fold), miRNA-27b-5p (-7.2-fold), and miRNA-200b-3p (-6-fold) (Supplemental Table S4).

Sixty differentially expressed miRNAs were identified in the differentiated ECL cells from the three ASC-tested sources. Common miRNAs across the three sources were displayed using Venn diagrams (Figure 2B). Notably, 17 were common between differentiated ECL cells from sASCs of lean and obese subjects (Figure 2B), and two of them were in the top five of both metabolic conditions (miRNA-181a-2-3p and miRNA-542-5p). On the other hand, only one miRNA was common to differentiated ECL cells from vASCs and sASCs of the same obese donor (miRNA-1290). Similarly, only one miRNA was shared by differentiated ECL cells from vASCs and sASCs of lean subjects (miRNA-222-5p) (Figure 2B).

Importantly, miRNA-27b-5p was present in all three conditions as a significantly downregulated miRNA after differentiation into ECL cells. No miRNA showed an opposite expression pattern between different conditions. Additionally, it should be noted that we found the same miRNAs in ECL cells differentiated from sASCs of lean and obese subjects. The miRNA-378-3p family comprises miRNA-378a-3p, miRNA-378c, miRNA-378i, and miRNA-378f in differentiated ECL cells from sASCs of lean subjects and miRNA-378a-3p, miRNA-378c, and miRNA-378f in differentiated ECL cells from sASCs of obese individuals. In differentiated ECL cells from sASCs of both lean and obese subjects, we identified miRNA-143/145 and miRNA-424/503 clusters. Additionally, in differentiated ECL cells from sASCs of obese individuals, miRNA-23b/27b/24-1 and DLK1-DIO3 miRNA clusters were found. Importantly, two of the miRNAs selected for further validation were present within these clusters. Interestingly, in cells from visceral tissue, no cluster or family of miRNAs was identified in the differentiated ECL cells.

### 3.3. MiRNA-424-5p, miRNA-378a-3p, and miRNA-29b-3p Regulate ASCs’ Angiogenic Differentiation

We have previously demonstrated that miRNA-145-5p regulates sASCs’ differentiation into ECL cells and induces vascular network formation [30]. Herein, we have identified additional miRNAs that contribute to the differentiation of ASCs into ECL cells. To investigate the main molecular mechanisms and functions of miRNAs, we designed a workflow of in silico analyses and in vitro validations to explore the role of these miRNAs and their putative direct targets in the angiogenic differentiation of ASCs.

Six of the most significantly regulated miRNAs were selected as potential candidates for further study: miRNA-27b-5p, miRNA-424-5p, miRNA-378a-3p, miRNA-29b-3p, miRNA-146a-5p, and miRNA-214-5p. MiRNA-27b-5p was selected because it was significantly downregulated in the differentiated ECL cells from the three original ASCs investigated (Supplemental Tables S2–S4). Both miRNA-146a-5p and miRNA-378a-3p were in the top 10 most significantly upregulated miRNAs with high average expression in differentiated ECL cells, whereas miRNA-424-5p was one of the top five differentially downregulated miRNAs (Supplemental Tables S2 and S3). MiRNA-214-5p and miRNA-29b-3p were selected because they were the most upregulated miRNAs in differentiated ECL cells from vASCs and sASCs of obese and lean subjects, respectively (Supplemental Tables S3 and S4).

**Figure 3:**
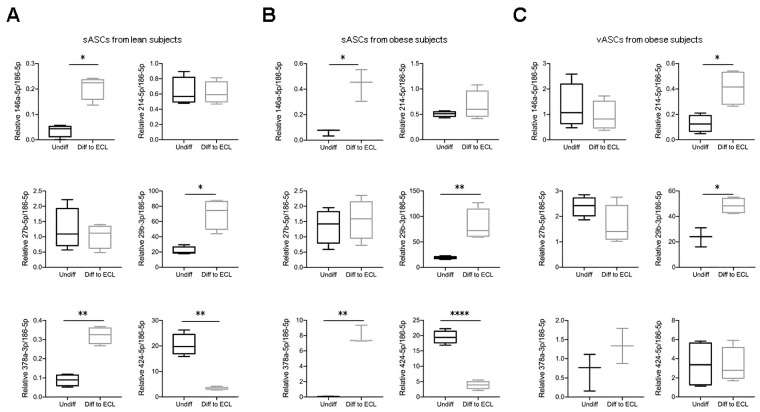
Differentially expressed miRNAs in ASCs differentiated into ECL cells. ASCs were treated for nine days with basal DMEM or DMEM supplemented with bFGF, and hsa-miRNA-146a-5p, hsa-miRNA-214-5p, hsa-miRNA-27b-5p, hsa-miRNA-29b-3p, hsa-miRNA-378a-3p, and hsa-miRNA-424-5p expression was quantified by qRT-PCR in (**A**) sASCS from lean individuals, (**B**) in sASCS from obese individuals, and (**C**) in vASCs from obese individuals. Cells in B and C are from the same donors. Results are shown as the arithmetic mean ± SD of six independent experiments, and statistical analysis was performed by ANOVA followed by Tukey’s post hoc test. * *p*-value < 0.05, ** *p*-value < 0.01, and **** *p*-value < 0.0001 (*n* = 6).

Different miRNAs were validated by qRT-PCR analysis. In sASCs differentiated into ECL cells from both lean and obese subjects, the same miRNAs were validated. MiRNA-146-5p (*p*-value < 0.05), miRNA-29b-3p (*p*-value < 0.05), and miRNA-378a-3p (*p*-value < 0.01 and *p*-value < 0.001) showed an increase, whereas miRNA-424-5p was downregulated (*p*-value < 0.01 and *p*-value < 0.0001, respectively (Figure 3A,B). On the other hand, in ECL cells from vASCs of obese subjects, two miRNAs were validated. Both miRNAs, miRNA-214-5p and miRNA-29b-3p, were upregulated (*p*-value < 0.05) (Figure 3C). It is important to note that vASCs from obese subjects showed high variability between samples, which could be attributed to individual obesity metabolic effects.

### 3.4. In Silico Analysis: miRNAs Target Angiogenesis-Associated Genes FGFR1, NRP2, MAPK1, and TGF-β2 in Differentiated ASCs into ECL Cells

Target genes of each miRNA were identified using four different online target predicting software programs, identifying a total of 154 target genes. The intersection targets for each miRNA were displayed using Venn diagrams (Figure S1). Common targets across all databases for each miRNA are presented in Supplemental Table S5. To restrict the number of genes for in vitro validation, we carried out literature searches to confirm the contribution of selected targets to angiogenesis [32,33,34,35,36,37,38,39,40,41]. Only genes with biological meaning related to angiogenesis, neovessel formation, or cell differentiation were selected for further validation (Supplemental Table S5). However, none of the predicted target genes for miRNA-146a-5p and miRNA-214-5p were related to angiogenesis; therefore, we did not investigate these two miRNAs any further. Specifically, we analyzed the effects of miRNA-424-5p on FGFR1 and NRP2 and miRNA-378a-3p on MAPK1 in sASCs independent of the BMI status of subjects and the effects of miRNA-29b-3p on TGF-*β*2 in sASCs from lean donors and in sASCs/vASCs from obese subjects.

**Figure 4:**
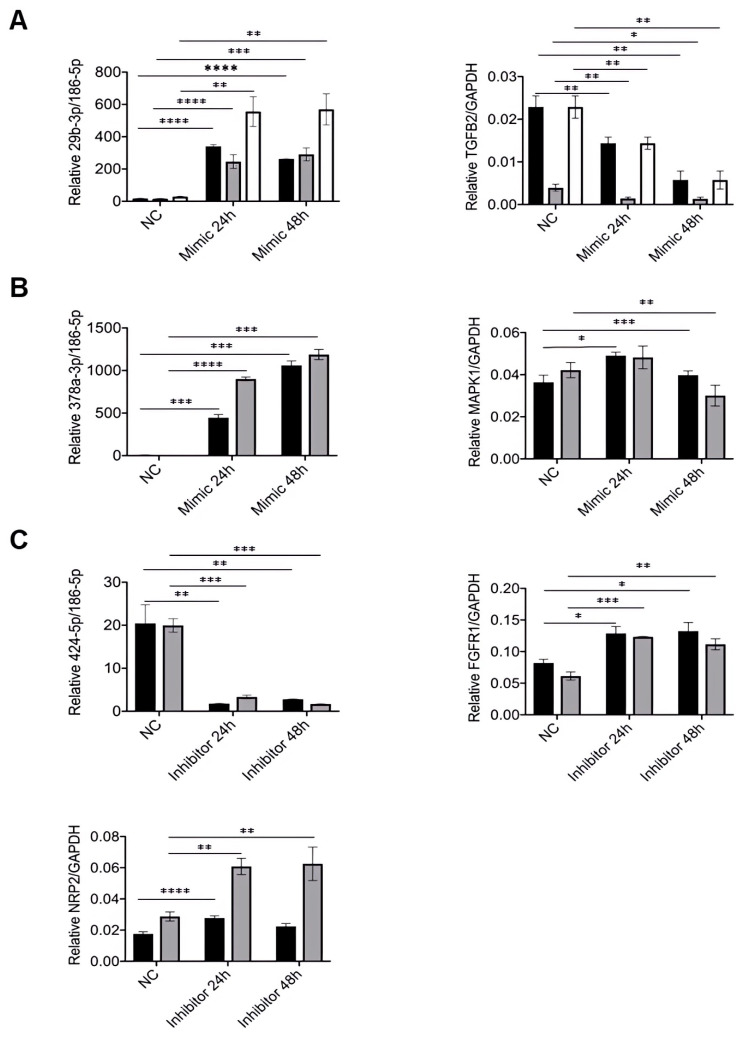
Effects of miRNAs on their direct target genes that mediate ASCs’ differentiation into ECL cells. ASCs were treated with the mimic or inhibitor negative control (NC), mimics or inhibitors of significant miRNAs for 24 h and 48 h. The relative miRNA expression as well as the regulation of the direct target gene were quantified by qRT-PCR. sASCs from lean individuals are shown in white columns, sASCs from obese subjects are shown in black columns, and vASCs from the same obese subject are shown in gray columns. (**A**) Relative miRNA-29b-3p expression and *TGF-β2* regulation after ASC treatment with the miRNA-29b-3p mimic. (**B**) Relative miRNA-378a-3p expression and *MAPK1* regulation after ASC treatment with the miRNA-378a-3p mimic. (**C**) Relative miRNA-424-5p expression and, *FGFR1* and *NRP2* regulation after ASC treatment with the miRNA-424-5p inhibitor. Results are shown as the arithmetic mean ± SD of six independent experiments, and statistical analysis was performed by ANOVA followed by Tukey’s post hoc test. * *p*-value < 0.05, ** *p*-value < 0.01, *** *p*-value < 0.001, and **** *p*-value < 0.0001 (*n* = 6).

As shown in Figure 4A, after mimic transfection, the expression of miRNA-29b-3p was significantly upregulated (*p*-value < 0.0001, *p*-value < 0.01, and *p*-value < 0.01, respectively) in all ASC types. Moreover, this upregulation resulted in reduced expression of its direct target TGF-*β*2 (*p*-value < 0.05 and *p*-value < 0.01) after 24 h of transfection and was maintained for 48 h, also in all tissue samples (Figure 4A). Similarly, miRNA-378a-3p mimic transfection in sASCs from lean and obese subjects significantly upregulated miRNA-378a-3p expression (*p*-value < 0.001 and *p*-value < 0.0001, respectively) at 24 h and 48 h (Figure 4B). However, significant downregulation of the expression of its direct target MAPK1 was only observed 48 h post-transfection, while a mild increase was observed at 24 h post-transfection (Figure 4B). Finally, in sASCs from lean and obese individuals, miRNA-424-5p inhibition at 24 h and 48 h (*p*-value < 0.01 and *p*-value < 0.001, respectively) (Figure 4C) resulted in increased expression of both FGFR1 (*p*-value < 0.05 and *p*-value < 0.001, respectively) and NRP2 (*p*-value < 0.0001 and *p*-value < 0.01, respectively) at 24 h and 48 h post-transfection (Figure 4C). However, NRP2 was more regulated in sASCs from obese subjects than in sASCs from lean individuals.

**Figure 5:**
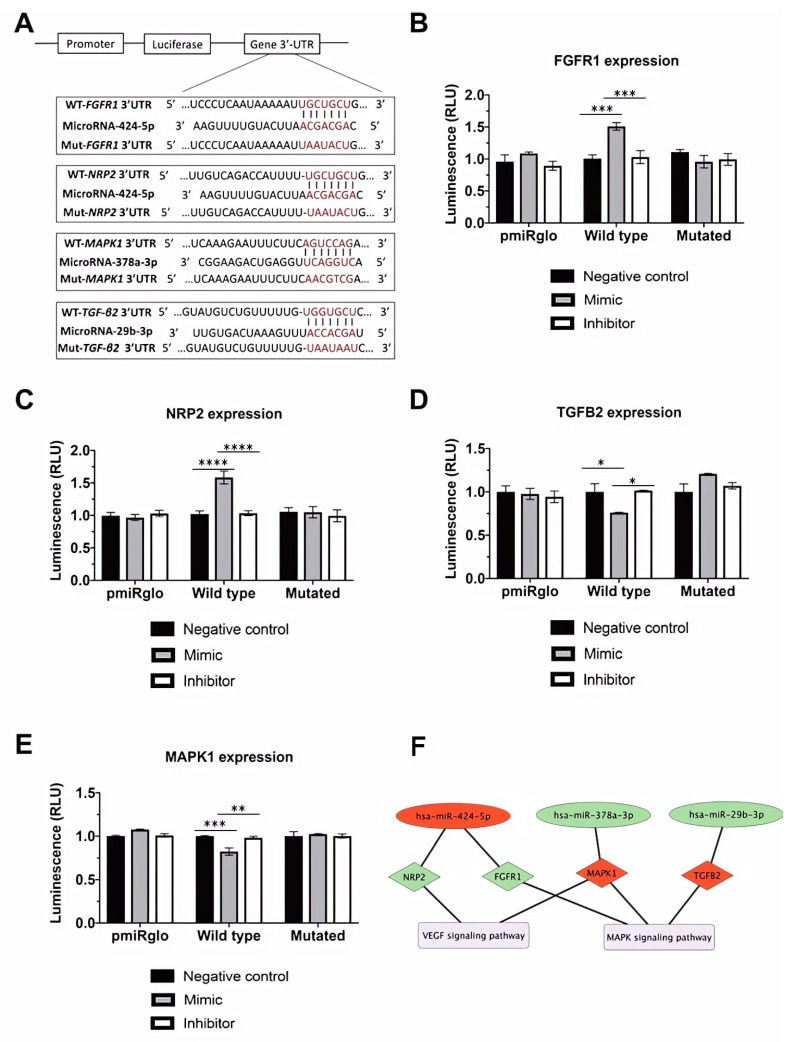
*FGFR1, MAPK1, NRP2*, and *TGF-β2* are direct targets of miRNA-29b-3p, miRNA-378a-3p, and miRNA-424-5p. (**A**) Bioinformatics-based prediction of the miRNA-424-5p, miRNA-378a-3p, and miRNA-29b-3p target sites in the 3′-UTR of *FGFR1, MAPK1, NRP2,* and *TGF-β2* mRNA. Red letters indicate the mutated sites in the miRNA seed region. (**B**) Relative luciferase activities 36 h after cotransfection of HeLa cells with miRNA-424-5p mimic, inhibitor, or negative control, and a luciferase reporter vector containing the wild-type or mutated 3′-UTR of *FGFR1*. (**C**) Relative luciferase activities 36 h after cotransfection of HeLa cells with miRNA-424-5p mimic, inhibitor, or negative control, and a luciferase reporter vector containing the wild-type or mutated 3′-UTR of *NRP2*. (**D**) Relative luciferase activities 36 h after cotransfection of HeLa cells with miRNA-378a-3p mimic, inhibitor, or negative control, and a luciferase reporter vector containing the wild-type or mutated 3′-UTR of *MAPK1.* (**E**) Relative luciferase activities 36 h after cotransfection of HEK 293 cells with miRNA-29b-3p mimic, inhibitor, or negative control, and a luciferase reporter vector containing the wild-type or mutated 3′-UTR of *TGF-β2*. Results are shown as the arithmetic mean ± SD of three independent experiments, and statistical analysis was performed by ANOVA followed by Tukey’s post hoc test. * *p*-value < 0.05, ** *p*-value < 0.01, *** *p*-value < 0.001, and **** *p*-value < 0.0001 (*n* = 3). (**F**) Regulation of the angiogenic differentiation of ASCs. KEGG and PANTHER analysis predicted that the represented miRNAs are targeting the genes associated with the pathways, as shown using Cytoscape software v3.9.0. The miRNAs are shown in red (downregulated) or green ellipses (upregulated), with black lines indicating the targeted upregulated (green) or downregulated (red) genes represented in diamonds, and the pathways involved in the mechanism are depicted by purple nodes.

Finally, we determined the direct binding between miRNAs and the predicted target site by performing a luciferase reporter assay. We analyzed the relative expression of miRNAs of interest in HeLa and HEK 293 cell lines. Gene expression assays showed that the relative expression of miRNA-378a-3p (Figure S2a) and miRNA-424-5p (Figure S2b) in HeLa cells was similar to that in sASCs. Even if relative miRNA-29b-3p expression was higher in HEK 293 cells than in sASCS cells, the difference was not statistically significant (Figure S2c). We determined whether miRNA-424-5p, miRNA-378a-3p, and miRNA-29b-3p had direct binding to their predicted target sites in the FGFR1, MAPK1, NRP2, and TGF-*β*2 3′-UTRs (Figure 5A). Luciferase reporter plasmids containing either the wild-type or mutated miRNA binding site in the FGFR1, MAPK1, NRP2, and TGF-*β*2 3′-UTR regions were cotransfected with mimic, inhibitor, or negative controls. Transfection of the miRNA-424-5p mimic resulted in increased luciferase activity of the wild-type reporter vector but not the mutated reporter vector for FGFR1 (*p*-value < 0.001 negative control vs. mimic and *p*-value < 0.001 mimic vs. inhibitor) (Figure 5B) and NRP2 (*p*-value < 0.0001 negative control vs. mimic and *p*-value < 0.0001 mimic vs. inhibitor) (Figure 5C). On the other hand, the overexpression of miRNA-378a-3p resulted in reduced luciferase activity of the wild type but not the mutated reporter vector (*p*-value < 0.001 negative control vs. mimic and *p*-value < 0.01 mimic vs. inhibitor) (Figure 5D), an effect that has also been detected for miRNA-29b-3p/TGF-*β*2 interaction (*p*-value < 0.01 negative control vs. mimic and *p*-value < 0.001 mimic vs. inhibitor) (Figure 5E). Thus, the 3′-UTR of FGFR1 and NRP2, MAPK1, and TGF-*β*2 are direct targets of miRNA-424-5p, miRNA-378a-3p, and miRNA-29b-3p, respectively.

Importantly, we show for the first time that miRNA-29b-3p upregulation contributes to ASCs’ differentiation into ECL cells by directly targeting TGF-*β*2 in both sASCS and vASCS. Moreover, our results reveal that, independent of sASC origin, the upregulation of miRNA-378a-3p and the downregulation of miRNA-424-5p inhibit MAPK1 and overexpress FGFR1 and NRP2, respectively (Figure 5F).

## 4. Discussion

AT angiogenesis is at the heart of AT expandability. Given the multiple, pivotal roles of the AT vasculature, impairments in angiogenic capacity may underlie obesity-associated diseases such as diabetes and cardio-metabolic disease. ASCs resident in AT have the capacity to differentiate into multiple cell lineages [42], including ECs [21,43,44], promoting angiogenesis. Here, we have investigated the epigenetic regulation of ASCs’ differentiation into ECL cells by searching their specific miRNA profiles with respect to their fat tissue depot and metabolic condition. The present study compares, for the first time, miRNA profiles between sASCs and vASCs from the same obese donor after endothelial differentiation.

Obesity impairs ASCs’ proliferation potential and survival. However, there are controversies between different studies. While Frazier et al. [45] found that BMI affects the proliferation rates of ASCs, no significant differences were observed by Todoya et al. [12]. In our study, we found that obesity did not modify the proliferation rates of ASCs. However, after bFGF treatment, significant differences in growth rates depending on fat depot location were found. sASCs from lean and obese subjects showed a doubling time of nine days. However, in vASCs from obese patients, bFGF had no significant effect on growth kinetics. These results are in line with those of Khan et al. [44] and Ma et al. [46]. The novelty of our results is that these studies only used sASCs, whereas for the first time, we demonstrate that bFGF does not change vASCs’ proliferation rates. Thus, we show that sASCs from different lean and obese subjects respond to bFGF treatment more similarly than ASCs from the same obese subjects but originating from different fat depots (sASCs and vASCs), regarding their induced proliferation rates.

The expansion of AT is functionally and necessarily related to angiogenesis. AT is a highly vascularized tissue with high angiogenic capacities. The cells that form this tissue are surrounded by an extensive capillary network, which requires constant regulation by several angiogenic modulators [47,48]. The crosstalk and cell-to-cell interactions between ASCs and ECs involve numerous paracrine factors associated with angiogenesis and/or cell differentiation, which underlie the changes in vascularization seen in obesity [47,49]. Recent publications suggest that miRNAs play a pivotal role in adipocyte differentiation [50,51,52]. Therefore, we believe that ASCs’ differentiation into ECL cells represents the perfect method to improve angiogenesis. We have previously demonstrated that bFGF successfully induces sASCs’ differentiation into ECL cells by regulating miRNA/mRNA interactions. MiRNA expression profiles seem to be fat-depot specific and are correlated with obesity and metabolic disorders [50,53]. Toyoda et al. characterized and compared ASCs from subcutaneous AT and omental AT from lean subjects and demonstrated that sASCs have a higher differentiation capacity into different lineages than omental ASCs [12]. Moreover, it has been reported that subcutaneous AT and sASCs have a higher pro-angiogenic capacity compared to visceral AT and vASCs [26]. We have demonstrated that sASCs, regardless of the BMI status of the individual, are enriched in miRNAs involved in the differentiation capacity of ASCs, in contrast to vASCs. Moreover, miRNA characterization reinforced these results; sASCs from lean and obese subjects had the same miRNA levels as well as vascular EC marker expression, whereas they differed in vASCs. However, miRNA-378a-3p levels were higher in sASCs and vASCs from obese subjects compared to sASCS from lean subjects. This suggests that this miRNA may be regulated by the metabolic conditioning of obesity. It should also be noted that even if obesity did not influence proliferation, it did influence miRNA expression. In sASCS from lean subjects, the majority of the miRNAs were upregulated after bFGF treatment, whereas in sASCs and vASCs from the same obese donor, the majority of the miRNAs were downregulated. So, these results suggest that obesity did not influence the quantity of expressed miRNAs, as sASCs from lean and obese subjects expressed the same number of miRNAs, but it did influence the type of miRNA regulation.

Using a miRNA-based sequencing approach, we identified miRNAs associated with ASCs’ differentiation into ECL cells after nine days of exposure to bFGF. From our reported miRNAs, all three miRNAs (29b-3p, 378a-3p, and 424-5p) target downstream genes of the MAPK pathway, which induces human-induced pluripotent stem cells to differentiate into ECs [54]. MiRNA-424-5p regulates cell-intrinsic angiogenic responses such as proliferation, migration, and differentiation of ECs and cancer cells. The overexpression of miRNA-424-5p reduces cell proliferation, migration, and invasion by regulating different genes [32,55,56,57]. Herein, we found that this miRNA is downregulated in sASCs differentiated into ECL cells and regulates ASCs’ differentiation through *FGFR1-* and *NRP2*-targeting. *FGFR1* is a tyrosine–kinase receptor selectively expressed on ECs both in vivo and in vitro. This receptor mediates the pro-angiogenic effects of bFGF [58,59]. miRNA-424-5p/*FGFR1* activation stimulates the angiogenic signal transduction pathways that induce the proliferation, migration, and differentiation of ECs [32]. In addition, *NRP2* is a cell surface transmembrane protein characterized as a VEGFR1 and VEGFR2 coreceptor. It is primarily expressed in the vascular system during VEGF signaling. EC-CD34+ tip cells express high levels of *NRP2*. Although it does not play a role in tip cell formation, it does support the formation of new vessel sprouts [41,60]. Therefore, this is the first report describing the role played by *FGFR1* and *NRP2* in ASCs’ differentiation into ECL cells.

MiRNA-378a-3p has been described to contribute to the formation of vascular branches in ECs by targeting FGF in skeletal muscle [61]. Moreover, this miRNA participates in cell differentiation processes, and its overexpression contributes to ASCs’ differentiation into smooth muscle cells, similar to *TGF-β1* treatment [62]. Regarding the differentiation mechanism, accumulating evidence has previously demonstrated the importance of miRNA-378a-3p in cell differentiation. In this article, we demonstrate that the upregulation of this miRNA plays a key role in sASCs’ differentiation into ECL cells and contributes to ASCs’ angiogenic differentiation by directly targeting *MAPK1*. EKR1/2 is a kinase that plays a prominent role in EC angiogenic functions by regulating their proliferation and migration. In particular, ERK2, also known as *MAPK1*, is the primary driver of endothelial proliferation and regulates eNOS expression, while migration is controlled by both isoforms [34,35].

Finally, for the first time, we describe a specific miRNA that regulates ECL cell differentiation in both sASCs and vASCs. The literature indicates that the role of miRNA-29b in cell growth and apoptosis is cell-type-specific. MiRNA-29b-3p is involved in EC migration, proliferation, and tube formation [63,64]. Here, we have demonstrated that the upregulation of miRNA-29b-3p directly targets *TGF-β2,* inhibiting its translation and promoting ASCs’ ECL cell differentiation. *TGF-β2* is one of the three isoforms of TGF-*β*. Different molecular functions, such as cell differentiation of mesenchymal stem cells, growth of vascular ECs, and angiogenesis, are influenced by TGF-*β* [65]. KEGG analysis revealed that the MAPK and VEGF signaling pathways associated with *TGF-β2* play regulatory roles in endothelial differentiation.

This study is not free of certain limitations. Subcutaneous and visceral AT were obtained from the same donor, but we could not obtain visceral AT from lean individuals. Indeed, visceral AT is a difficult tissue to obtain because it is necessary to undergo bypass gastric surgery, and the donors of lean subcutaneous tissue were individuals who underwent abdominal lipectomy only. However, our results reveal different expression profiles in ASCs from the same donors but with different origins.

## 5. Conclusions

In conclusion, we have revealed several miRNAs that are differentially expressed in ASC to ECL differentiation. Although each miRNA has many potential targets, which indicates that different signal transduction pathways can be regulated by the same miRNA and that several miRNAs regulate the same signaling pathway, we have identified specific effects of miRNas expressed during the differentiation programming of ASCs into ECL. We show for the first time that miRNA-29b-3p upregulation contributes to ASCs’ differentiation into ECL cells by directly targeting TGF-*β*2 in both sASCS and vASCS. Moreover, our results reveal that, independent of sASC origin, the upregulation of miRNA-378a-3p and the downregulation of miRNA-424-5p inhibit MAPK1 and overexpress FGFR1 and NRP2, respectively. We have also demonstrated that the adipose local depot affects the differentiation of resident ASCs through the expression of specific miRNAs. Our findings provide a new paradigm for the role of miRNAs in orchestrating ASCs’ differentiation into ECL cells, depending on ASCs’ adipose tissue depot.

## Data Availability

The datasets used and/or analyzed during the current study are available from the corresponding author upon reasonable request.

## Supplementary Materials

The following supporting information can be downloaded at: https://www.mdpi.com/article/10.3390/cells13060513/s1, Figure S1: Predicted targeted genes of significantly expressed miRNAs. Venn diagram of the candidate target genes predicted by four databases; Figure S2: miRNAs’ relative expression in HeLa and HEK 293 cell lines; Table S1: Clinical characteristics of the subjects who participate in the study; Table S2: Differential expression of miRNAs in ECL cells differentiated from sASCs of lean individuals versus the parental undifferentiated sASCs (significantly changed); Table S3: Differential expression of miRNAs in ECL cells differentiated from ASCs of obese individuals versus the parental undifferentiated sASCs (significantly changed); Table S4: Differential expression of miRNAs in ECL cells differentiated from vASCs of obese individuals versus the parental undifferentiated vASCs (significantly changed); Table S5: List of the candidate target genes predicted by four databases and their relation to angiogenesis and neovessel formation.
