# Supplementary material for: Differentiation of Adipose Tissue Mesenchymal Stem Cells into Endothelial Cells Depends on Fat Depot Conditions: Regulation by miRNA
Source: Cells. 2024 Mar 14;13(6):513. doi: 10.3390/cells13060513 (PMC10969675; doi:10.3390/cells13060513)
Supplement: Supplementary file 1 [file cells-13-00513-s001.zip › cells-2883744-supplementary.pdf]

Supplemental material

The differentiation of adipose tissue mesenchymal stem cells into endothelial cells depends on fat depot and metabolic conditions: regulation by miRNA

Gemma Arderiu[*,+,1,2], Anna Civit-Urgell[*,1], Fabrizio Moscatiello[3], Lina Badimon[1,2]

[1] Cardiovascular-Program; Institut de Recerca de l'Hospital de la Santa Creu i Sant Pau. IIB-Sant Pau Barcelona, Spain; [2] Ciber CV, Instituto Carlos III, Madrid, Spain; [3]Centro Médico Teknon, Grupo Quiron Salut, Barcelona, Spain.

*Contributed equally to this work; +corresponding author

Short Title: MicroRNA sequencing depends on ASCs fat depot.

Correspondence author: Gemma Arderiu
Cardiovascular-Program
Institut de Recerca de Sant Pau- IIB Sant Pau
C/ Sant Antoni Mª Claret 167, 08025 Barcelona, Spain
Tel: 34 93 556 58 80
ORCID: 0000-0001-8598-4106
E-mail: garderiu@santpau.cat

Supplementary figures:

Supplementary Figure S1: Predicted targeted genes of significantly expressed miRNAs. Venn diagram of the candidate target genes predicted by four databases. Using Cytoscape software v3.9.0, miRNAs are shown in red ellipses (down-regulated) or green ellipses (up-regulated) with the black lines to the targeted gene in green rectangles (up-regulated) or red rectangles (down-regulated) nodes. Targeted genes in yellow are suggested for further validation due to their angiogenic properties.

Supplementary Figure S2: miRNAs relative expression in HeLa and HEK 293 cell lines. **(A)** Relative expression of miRNA-378a-3p in HeLa cells compared to sASCs, **(B)** Relative expression of miRNA-424-5p in HeLa cells compared to sASCs, and **(C)** Relative expression of miRNA-29b-3p in HEK 293 compared to sASCS. No statistically significant differences were found in any comparison.

Supplementary Figure S1

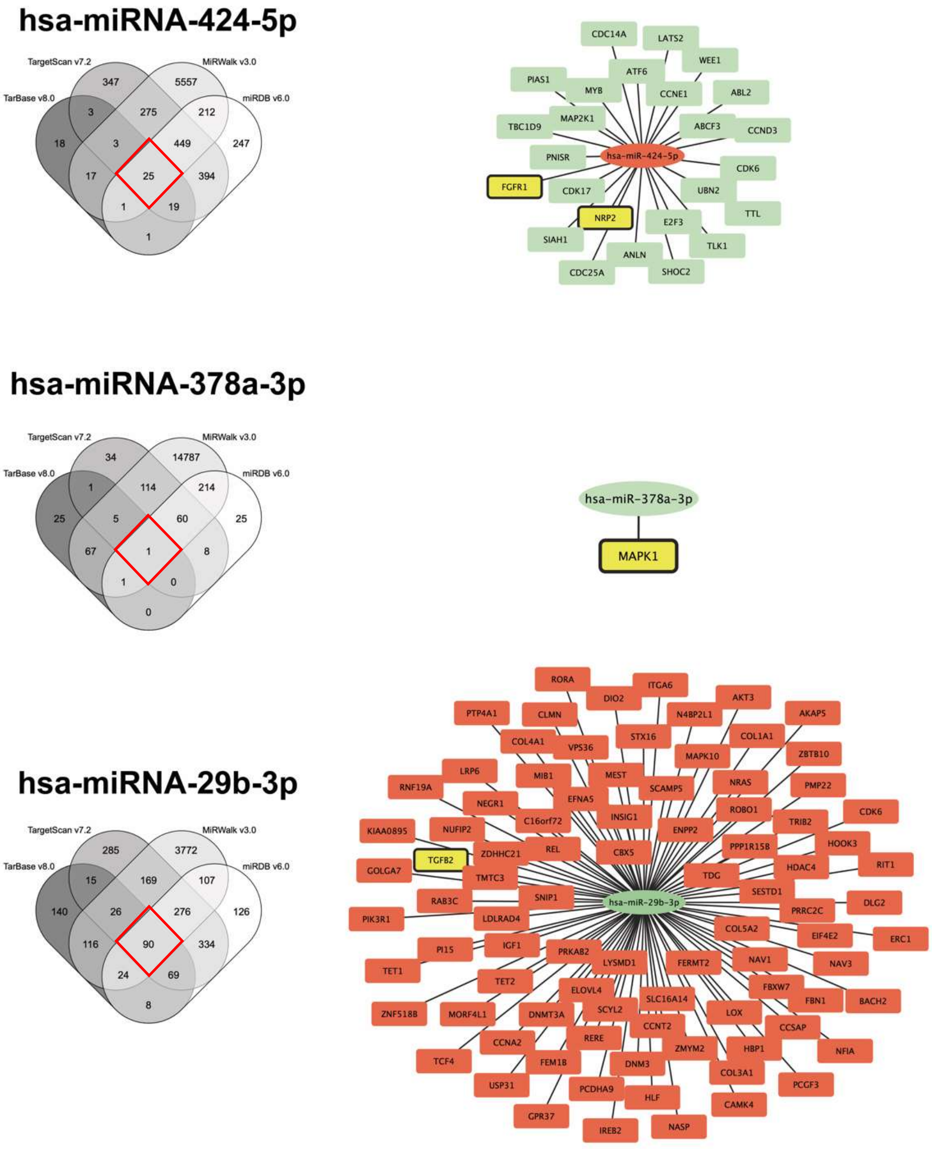

Supplementary Figure S2

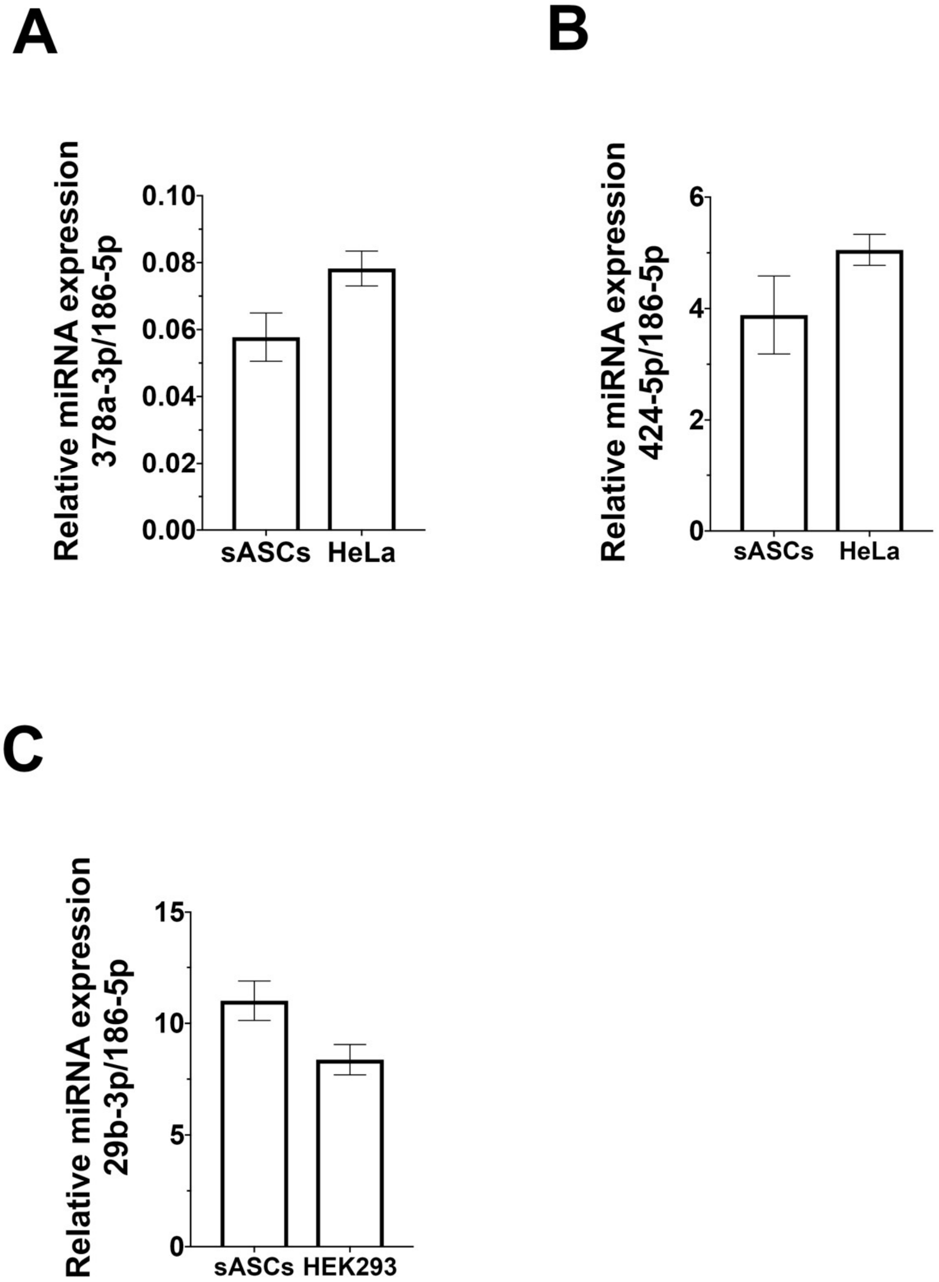

Supplementary Table S1: Source of the bio-banked stem cells used in this study.

**SUBJECTS**	**TISSUE SAMPLE**	**SURGERY**	**AGE**	**SEX**	**BMI** (KG/M^2)	**WEIGHT** (KG)
**N=6**	Subcutaneous	Abdominal lipectomy	39,2±11,5	4 Women<br>2 Men	23,97±3,60	69±14,23
**N=6**<br>**N=6**	Subcutaneous and omentum	Gastric by-pass and cholecystectomy	50,8±9,2	3 Women<br>3 Men	41,69±7,56	115,03±26,52

Data are presented as the mean±SD.

UP-REGULATED miRNAs in ECL cells from lean sACSs versus original lean sASCs

microRNA	Fold change	p-value	Average expression
hsa-miRNA-29b-3p	1705,73	3,14E-05	3,21
hsa-miRNA-146a-5p	1553,38	4,27E-04	5,69
hsa-miRNA-378c	1095,23	3,81E-06	4,86
hsa-miRNA-92a-1-5p	774,20	9,01E-05	3,62
hsa-miRNA-378a-3p	648,58	1,32E-06	7,00
hsa-miRNA-422a	452,75	1,23E-03	3,73
hsa-miRNA-199b-5p	432,59	1,11E-05	1,94
hsa-miRNA-708-5p	394,28	1,55E-04	6,78
hsa-miRNA-378i	295,13	1,73E-03	3,10
hsa-miRNA-222-5p	286,92	2,50E-03	2,65
hsa-miRNA-378f	182,68	6,30E-03	3,66
hsa-miRNA-4284	137,45	2,62E-05	5,69
hsa-miRNA-31-3p	116,87	9,46E-06	5,91
hsa-miRNA-7110-5p	112,27	1,52E-04	3,98
hsa-miRNA-29b-1-5p	81,15	2,38E-07	7,62
hsa-miRNA-221-5p	79,35	2,87E-03	4,24
hsa-miRNA-584-5p	79,07	4,16E-03	3,85
hsa-miRNA-25-5p	78,19	2,84E-03	3,88

DOWN-REGULATED miRNAs in ECL cells from Lean sACSs versus original lean sASCs

microRNA	Fold change	p-value	Average expression
hsa-miRNA-27b-5p	0,011871997	2,27E-03	4,51
hsa-miRNA-585-5p	0,009948639	5,09E-03	3,81
hsa-miRNA-675-5p	0,00481043	1,25E-03	2,27
hsa-miRNA-424-3p	0,004027724	2,85E-07	8,24
hsa-miRNA-503-5p	0,004023201	6,51E-08	7,40
hsa-miRNA-424-5p	0,002000294	1,41E-05	2,42
hsa-miRNA-143-3p	0,0019046	1,62E-07	9,61
hsa-miRNA-145-5p	0,001337622	1,02E-08	10,77
hsa-miRNA-542-5p	0,000864988	2,90E-05	3,09
hsa-miRNA-181a-2-3p	0,000644403	8,53E-06	5,09

Supplementary Table S2: Differential expression of miRNAs in ECL cells differentiated from sASCs of lean individuals versus the parental undifferentiated sASCs (significantly changed).

UP-REGULATED miRNAs in ECL cells from obese sACSs versus original obese sASCs

microRNA	Fold change	p-value	Average expression
hsa-miRNA-422a	441,09	1,28E-03	3,73
hsa-miRNA-199b-5p	283,99	3,44E-05	1,94
hsa-miRNA-146a-5p	211,64	8,11E-03	5,69
hsa-miRNA-378c	149,27	4,87E-04	4,86
hsa-miRNA-92a-1-5p	133,83	2,79E-03	3,62
hsa-miRNA-7641	130,91	1,57E-03	6,24
hsa-miRNA-3175	125,42	2,14E-03	3,00
hsa-miRNA-378a-3p	106,16	2,24E-04	7,00
hsa-miRNA-378f	105,69	1,37E-02	3,66
hsa-miRNA-708-5p	82,31	3,87E-03	6,78
hsa-miRNA-25-5p	71,79	3,37E-03	3,88
hsa-miRNA-218-5p	65,32	7,65E-04	1,67

DOWN-REGULATED miRNAs in ECL cells from obese sACSs versus original obese sASCs

microRNA	Fold change	p-value	Average expression
hsa-miRNA-98-5p	0,01455	5,21E-04	3,21
hsa-miRNA-493-3p	0,01330	8,11E-04	5,45
hsa-miRNA-628-3p	0,01175	4,14E-03	3,30
hsa-miRNA-1296-5p	0,01005	1,09E-02	3,12
hsa-miRNA-503-5p	0,01001	2,52E-06	7,40
hsa-miRNA-433-3p	0,00906	3,26E-05	5,95
hsa-miRNA-424	0,00866	2,43E-07	2,19
hsa-miRNA-424-3p	0,00748	2,87E-06	8,24
hsa-miRNA-27b-5p	0,00723	7,90E-04	4,51
hsa-miRNA-329-3p	0,00629	5,96E-04	3,80
hsa-miRNA-495-3p	0,00564	1,91E-03	4,51
hsa-miRNA-503-3p	0,00563	5,74E-06	1,94
hsa-miRNA-1185-1-3p	0,00562	6,65E-05	4,76
hsa-miRNA-487a-5p	0,00510	3,93E-04	3,91
hsa-miRNA-664a-5p	0,00498	1,48E-05	2,56
hsa-miRNA-143-5p	0,00435	1,07E-06	1,89
hsa-miRNA-154-5p	0,00403	7,83E-04	3,70
hsa-miRNA-377-5p	0,00379	3,31E-05	3,21
hsa-miRNA-126-3p	0,00368	1,68E-04	5,60
hsa-miRNA-210-5p	0,00331	6,38E-06	2,49
hsa-miRNA-10b-3p	0,00302	4,28E-05	2,01
hsa-miRNA-145-5p	0,00289	1,68E-07	10,77

hsa-miRNA-493-5p	0,00275	8,21E-04	4,04
hsa-miRNA-140-5p	0,00270	1,35E-04	4,81
hsa-miRNA-6511a-3p	0,00245	4,33E-06	3,26
hsa-miRNA-181d-5p	0,00241	1,85E-06	3,91
hsa-miRNA-487a-3p	0,00203	1,61E-04	4,77
hsa-miRNA-210-3p	0,00189	3,29E-07	7,70
hsa-miRNA-143-3p	0,00176	1,24E-07	9,61
hsa-miRNA-23b-5p	0,00135	1,41E-04	4,58
hsa-miRNA-424-5p	0,00060	5,83E-07	2,42
hsa-miRNA-542-5p	0,00060	1,31E-05	3,09
hsa-miRNA-1290	0,00021	8,97E-06	3,81
hsa-miRNA-181a-2-3p	0,00005	2,04E-08	5,09

Supplementary Table S3: Differential expression of miRNAs in ECL cells differentiated from ASCs of obese individuals versus the parental undifferentiated sASCs (significantly changed).

UP-REGULATED miRNAs in ECL cells from obese vACSs versus original obese vASCs

microRNA	Fold change	p-value	Average expression
hsa-miRNA-214-5p	178,40	5,36E-03	4,78
hsa-miRNA-222-5p	70,00	2,04E-02	2,65

DOWN-REGULATED miRNAs in ECL cells from obese vACSs versus original obese vASCs

microRNA	Fold change	p-value	Average expression
hsa-miRNA-200b-3p	0,015	6,87E-05	1,34
hsa-miRNA-27b-5p	0,007	6,96E-04	4,51
hsa-miRNA-30c-2-3p	0,005	3,57E-04	3,24
hsa-miRNA-1290	0,003	1,28E-03	3,81

Supplementary Table S4: Differential expression of miRNAs in ECL cells differentiated from vASCs of obese individuals versus the parental undifferentiated vASCs (significantly changed).

miRNA	Target genes	Angiogenesis association
hsa-miRNA-424-5p	*ABCF3, ABL2, ANLN, ATF6, CCND3, CCNE1, CDC14A, CDC25A, CDK17, CDK6, E2F3, FGFR1, LATS2, MAP2K1, MYB, NRP2, PIAS1, PNISR, SHOC2, SIAH1, TBC1D9, TLK1, TTL, UBN2, WEE1*	Chamorro-Jorganes, A. *et al. (2011)* [1]. Alghamdi, A. A. A. *et al. (2020)*[2]. Zachary, I. *et al.* (2014) [3]. Dallinga, MG. *et al.* (2021) [4]. Geretti, E. *et al.* (2008) [5].
hsa-miRNA-378a-3p	*MAPK1*	Ricard, N. *et al.* (2019) [6]. Srinivasan, R. *et al.* (2009) [7]. Walker, AMN. *et al.* (2021) [8].
hsa-miRNA-29b-3p	*AKAP5, AKT3, BACH2, C16orf72, CAMK4, CBX5, CCNA2, CCNT2, CCSAP, CDK6, CLMN, COL1A1, COL3A1, COL4A1, COL5A2, DIO2, DLG2, DNM3, DNMT3A, EFNA5, EIF4E2, ELOVL4, ENPP2, ERC1, FBN1, FBXW7, FEM1B, FERMT2, GOLGA7, GPR37, HBP1, HDAC4, HLF, HOOK3, IGF1, INSIG1, IREB2, ITGA6, KIAA0895, LDLRAD4, LOX, LRP6, LYSMD1, MAPK10, MEST, MIB1, MORF4L1, N4BP2L1, NASP, NAV1, NAV3, NEGR1, NFIA, NRAS, NUFIP2, PCDHA9, PCGF3, PI15, PIK3R1, PMP22, PPP1R15B, PRKAB2, PRRC2C, PTP4A1, RAB3C, REL, RERE, RIT1, RNF19A, ROBO1, RORA, SCAMP5, SCYL2, SESTD1, SLC16A14, SNIP1, STX16, TCF4, TDG, TET1, TET2, TGF-β2, TMTC3, TRIB2, USP31, VPS36, ZBTB10, ZDHHC21, ZMYM2, ZNF518B*	Liao, K. H. *et al.* (2017) [9]. Di Bernardini, E. *et al.* (2014) [10].
hsa-miRNA-146a-5p	*APPL1, FBXW2, IRAK1, KLF7, LCOR, MYBL1, NOVA1, NUCKS1, RFX7, RIMS2, RUNX1T1, SAMD8, SCN3B, TDRKH, ZBTB2, ZNF512B, ZNRF3*	
hsa-miRNA-214-5p	*CISH*	

Genes marked in red are selected for validation due to their angiogenic properties.

Supplementary Table S5: List of the candidate target genes predicted by four databases and their relation to angiogenesis and neovessel formation.

References

1. Chamorro-Jorganes A, Araldi E, Penalva LOF, et al (2011) MicroRNA-16 and MicroRNA-424 regulate cell-autonomous angiogenic functions in endothelial cells via targeting vascular endothelial growth factor receptor-2 and fibroblast growth factor receptor-1. Arterioscler Thromb Vasc Biol 31:2595–2606. https://doi.org/10.1161/ATVBAHA.111.236521
2. Alghamdi AAA, Benwell CJ, Atkinson SJ, et al (2020) NRP2 as an Emerging Angiogenic Player; Promoting Endothelial Cell Adhesion and Migration by Regulating Recycling of α5 Integrin. Front Cell Dev Biol 8:1–16. https://doi.org/10.3389/fcell.2020.00395
3. Zachary I (2014) Neuropilins: role in signalling, angiogenesis and disease. Chem Immunol Allergy 99:37–70. https://doi.org/10.1159/000354169
4. Dallinga MG, Habani YI, Schimmel AWM, et al (2021) The Role of Heparan Sulfate and Neuropilin 2 in VEGFA Signaling in Human Endothelial Tip Cells and Non-Tip Cells during Angiogenesis In Vitro. Cells 10:. https://doi.org/10.3390/CELLS10040926
5. Geretti E, Shimizu A, Klagsbrun M (2008) Neuropilin structure governs VEGF and semaphorin binding and regulates angiogenesis. Angiogenesis 11:31–39. https://doi.org/10.1007/S10456-008-9097-1
6. Ricard N, Zhang J, Zhuang ZW, Simons M (2019) Isoform-Specific Roles of ERK1 and ERK2 in Arteriogenesis. Cells 9:1–17. https://doi.org/10.3390/cells9010038
7. Srinivasan R, Zabuawala T, Huang H, et al (2009) Erk1 and erk2 regulate endothelial cell proliferation and migration during mouse embryonic angiogenesis. PLoS One 4:. https://doi.org/10.1371/journal.pone.0008283
8. Walker AMN, Warmke N, Mercer B, et al (2021) Endothelial Insulin Receptors Promote VEGF-A Signaling via ERK1/2 and Sprouting Angiogenesis. Endocrinology 162:. https://doi.org/10.1210/ENDOCR/BQAB104
9. Liao KH, Chang SJ, Chang HC, et al (2017) Endothelial angiogenesis is directed by RUNX1T1-regulated VEGFA, BMP4 and TGF-β2 expression. PLoS One 12:1–19. https://doi.org/10.1371/journal.pone.0179758
10. Di Bernardini E, Campagnolo P, Margariti A, et al (2014) Endothelial Lineage Differentiation from Induced Pluripotent Stem Cells Is Regulated by MicroRNA-21 and Transforming Growth Factor β2 (TGF-β2) Pathways. J Biol Chem 289:3383. https://doi.org/10.1074/JBC.M113.495531
